# Supplementary material for: Acceptance of and Preference for COVID-19 Vaccination in India, the United Kingdom, Germany, Italy, and Spain: An International Cross-Sectional Study
Source: Vaccines (Basel). 2022 May 24;10(6):832. doi: 10.3390/vaccines10060832 (PMC9230582; doi:10.3390/vaccines10060832)
Supplement: Supplementary file 1 [file vaccines-10-00832-s001.zip › vaccines-1699218-supplementary.pdf]

Supplementary information - Questionnaire
COVID-19 vaccination willingness and acceptance

Background

The COVID-19 pandemic has caused profound effects on the societies and economies globally. Scientists and pharmaceutical companies have been working on vaccines for COVID-19. Reports on several vaccines have been released and some countries have initiated vaccination programmes. This survey aims to investigate acceptance and willingness to accept the COVID-19 vaccination among the public.

Preface

Thank you for your participation! This questionnaire consists of 3 sections of questions and can be completed in about 10 minutes. You can exit this questionnaire at any time. We will keep your information confidential.

Hello,

you are about to participate in our questionnaire. This questionnaire will include several parts and will only take no more than 10 minutes to complete. Do you consent to undergo the questionnaire?

- Agree
- Disagree

Are you above 18 years old?

- Yes
- No

This research does not include any funds from biomedical companies. If you have any questions, please feel free to send an email to t.liu.10@student.rug.nl.

Section 1. Demographic information

1. What is your gender?
   - Male
   - Female
   - Other
2. What is your age interval?
   - 18-25
   - 26-30
   - 31-35
   - 36-40
   - 41-45
   - 46-50
   - 51-55
   - 56-60
   - Above 60
3. What is your highest educational level?
   - Pre-primary education or primary school education
   - Middle school education
   - High school education
   - Vocational school education
   - Bachelor's degree
   - Master's degree
   - PhD degree
4. What country/region are you currently in?

5. What is the best description of your occupation or your working area?
   - Students
   - Managers
   - Professionals
   - Technicians and associate professionals
   - Clerical support workers
   - Service and sales workers
   - Skilled agricultural, forestry and fishery workers
   - Craft and related trade workers
   - Plant and machine operators and assemblers
   - Elementary occupations
   - Armed forces occupations
   - Other

6. How much is your annual salary? (Students can skip this question)
   - Under 10,000 USD
   - 10,001-20000 USD
   - 20,001-30,000 USD
   - 30,001-40,000 USD
   - 40,001-50,000 USD
   - 50,001-60,000 USD
   - 60,001-70,000 USD
   - Above 70,000 USD

7. How do you rate your willingness and acceptance to get COVID-19 vaccination?
   (Rate from totally unwilling 0 to totally willing 10, if the vaccines are generally available)

8. How do you rate your willingness and acceptance if your friends, family members or employers recommend that you do so?
   (Rate from totally unwilling 0 to totally willing 10, if th generally available)

9. Have you ever been infected with COVID-19?
   - Yes
   - No
   - Not to answer

10. Have your friends, family members, employers, neighbours, or anybody you know in your community been infected with COVID-19?
    - Yes
    - No
    - Not to answer

11. What is your marital status?
    - Single
    - Married
    - Divorced
    - Other
    - Not to answer

12. What's your major source of COVID-19 vaccine information?
    - Healthcare provider
    - CDC or public health department
    - News reports
    - Social media
    - Friends or family members
    - Employers
    - Pharmaceutical company advertisement
    - Other

Section 2. Choosing the best answer based on a scenario

Scenario#1

Suppose you are going to get vaccinated, and you are facing several choices of action. Please select the answer that you are most satisfied with？

Once you have completed this question, you can still click the back button to return to this page at any time, and you can change your answer at any time.

	Vaccine A	Vaccine B	Neither
Vaccinevarieties	Inactivated vaccines	Adenovirus vector vaccines	
Adverse effect	moderate	very mild	
Efficacy	85%	75%	
Time for the vaccine startingworking	10 days	5 days	Neither
The duration ofvaccine works	10 months	15 months	
The cost of vaccination	$0	$50	
	Select This	Select This	Select This

Scenario#2

Suppose you are going to get vaccinated, and you are facing several choices of action. Please select the answer that you are most satisfied with？

Once you have completed this question, you can still click the back button to return to this page at any time, and you can change your answer at any time.

	Vaccine A	Vaccine B	Neither
• Vaccinevarieties	mRNA vaccine	mRNA vaccine	
• Adverse effect	mild	moderate	
• Efficacy	65%	55%	
• Time for the vaccine startingworking	15 days	20 days	Neither
• The duration ofvaccine works	20 months	5 months	
• The cost of vaccination	$150	$0	
	Select This	Select This	Select This

Scenario#3

Suppose you are going to get vaccinated, and you are facing several choices of action. Please select the answer that you are most satisfied with？

Once you have completed this question, you can still click the back button to return to this page at any time, and you can change your answer at any time.

	Vaccine A	Vaccine B	Neither
	Inactivated vaccine	Adenovirus vector	
• Vaccinevarieties<br>• Adverse effect	mild	very mild	
• Efficacy	55%	85%	
• Time for the vaccine startingworking	20 days	10 days	Neither
• The duration ofvaccine works	15 months	5 months	
• The cost of vaccination	$100	$200	
	Select This	Select This	Select This

Scenario#4

Suppose you are going to get vaccinated, and you are facing several choices of action. Please select the answer that you are most satisfied with？

Once you have completed this question, you can still click the back button to return to this page at any time, and you can change your answer at any time.

	Vaccine A	Vaccine B	Neither
Vaccinevarieties	mRNA vaccine	Inactivated	Neither
Adverse effect	very mild	mild	
Efficacy	65%	85%	
Time for the vaccine startingworking	10 days	15 days	
The duration ofvaccine works	10 months	20 months	
The cost of vaccination	$100	$50	
	CBC_Fixed1	CBC_Fixed1	CBC_Fixed1
	Select This	Select This	Select This

Scenario#5

Suppose you are going to get vaccinated, and you are facing several choices of action. Please select the answer that you are most satisfied with？

Once you have completed this question, you can still click the back button to return to this page at any time, and you can change your answer at any time.

	Vaccine A	Vaccine B	Neither
Vaccinevarieties	mRNA vaccine	mRNA vaccine	
Adverse effect	mild	very mild	
Efficacy	65%	85%	
Time for the vaccine startingworking	5 days	20 days	Neither
The duration ofvaccine works	5 months	20 months	
The cost of vaccination	$200	$50	
	CBC_Random6	CBC_Random6	CBC_Random6
	Select This	Select This	Select This

Scenario#6

Suppose you are going to get vaccinated, and you are facing several choices of action. Please select the answer that you are most satisfied with？

Once you have completed this question, you can still click the back button to return to this page at any time, and you can change your answer at any time.

- Vaccinevarieties
- Adverse effect
- Efficacy
- Time for the vaccine startingworking
- The duration ofvaccine works
- The cost of vaccination

Vaccine A	Vaccine B	Neither
Adenovirus vector	mRNA vaccine	
mild	moderate	
95%	95%	
5 days	15 days	Neither
20 months	10 months	
$50	$100	
CBC_Random4	CBC_Random4	CBC_Random4
Select This	Select This	Select This

Scenario#7

Suppose you are going to get vaccinated, and you are facing several choices of action. Please select the answer that you are most satisfied with？

Once you have completed this question, you can still click the back button to return to this page at any time, and you can change your answer at any time.

	Vaccine A	Vaccine B	Neither
• Vaccinevarieties	Inactivated vaccine	Adenovirus vector	
• Adverse effect	very mild	moderate	
• Efficacy	75%	55%	
• Time for the vaccine startingworking	15 days	10 days	Neither
• The duration ofvaccine works	15 months	20 months	
• The cost of vaccination	$150	$200	
	CBC_Random5	CBC_Random5	CBC_Random5
	Select This	Select This	Select This

Scenario#8

Suppose you are going to get vaccinated, and you are facing several choices of action. Please select the answer that you are most satisfied with?

Once you have completed this question, you can still click the back button to return to this page at any time, and you can change your answer at any time.

	Vaccine A	Vaccine B	Neither
• Vaccinevarieties	Adenovirus vector	mRNA vaccine	
• Adverse effect	very mild	moderate	
• Efficacy	65%	95%	
• Time for the vaccine startingworking	5 days	15 days	Neither
• The duration ofvaccine works	10 months	20 months	
• The cost of vaccination	$100	$150	
	CBC_Fixed2	CBC_Fixed2	CBC_Fixed2
	Select This	Select This	Select This

Scenario#9

Suppose you are going to get vaccinated, and you are facing several choices of action. Please select the answer that you are most satisfied with？

Once you have completed this question, you can still click the back button to return to this page at any time, and you can change your answer at any time.

	Vaccine A	Vaccine B	Neither
Vaccinevarieties	mRNA vaccine	Inactivated	Neither
Adverse effect	moderate	mild	
Efficacy	95%	65%	
Time for the vaccine startingworking	10 days	20 days	
The duration ofvaccine works	15 months	10 months	
The cost of vaccination	$150	$100	
	CBC_Random7	CBC_Random7	CBC_Random7
	Select This	Select This	Select This

Scenario#10

Suppose you are going to get vaccinated, and you are facing several choices of action. Please select the answer that you are most satisfied with？

Once you have completed this question, you can still click the back button to return to this page at any time, and you can change your answer at any time.

- Vaccinevarieties
- Adverse effect
- Efficacy
- Time for the vaccine startingworking
- The duration ofvaccine works
- The cost of vaccination

Vaccine A	Vaccine B	Neither
Inactivated	Adenovirus vector	
mild	moderate	
65%	85%	
10 days	15 days	Neither
10 months	15 months	
$50	$100	
CBC_Fixed3	CBC_Fixed3	CBC_Fixed3
Select This	Select This	Select This

Scenario#11

Suppose you are going to get vaccinated, and you are facing several choices of action. Please select the answer that you are most satisfied with？

Once you have completed this question, you can still click the back button to return to this page at any time, and you can change your answer at any time.

- Vaccinevarieties
- Adverse effect
- Efficacy
- Time for the vaccine startingworking
- The duration ofvaccine works
- The cost of vaccination

Vaccine A	Vaccine B	Neither
Adenovirus vector	Inactivated	
mild	very mild	
75%	95%	
15 days	5 days	Neither
10 months	20 months	
$0	$200	
CBC_Random8	CBC_Random8	CBC_Random8
Select This	Select This	Select This

Section 3. Psychological and behavioural part

This is the last part of our questionnaire. Thank you for your patience and support!

Past immunization behavior/ adverse events (**dichotomous, yes/no**)

- "I have once (or more than once) delayed getting a shot of vaccine for reasons other than illness or allergy?"
- "I have once decided not to get a shot of vaccine for reasons other than illness or allergy?"
- "I have events in the past that discouraged me from getting a vaccine(s) for myself or my families?" -> if yes, then next:
    - Did you ever experience an AEFI (adverse event following immunization)?

Cues to action (**dichotomous, yes/no**)

- "I am recommended by a doctor to get COVID-19 vaccination"
- "I am recommended by the local health board to get COVID-19 vaccination"
- "I heard that my friends/families are being vaccinated"

(Likert 7-point scales were applied to the following questions: totally disagree 1 - 7 totally agree)

Attitudes towards COVID-19 and COVID-19 vaccines

- (perceived knowledge) "I feel I get enough information about vaccines and their safety."
- (perceived knowledge) "The information I receive about vaccines from the vaccine program is reliable and trustworthy."
- (perceived susceptibility) "I think there is a great chance for me to be infected with COVID-19."
- (perceived severity) "I think a COVID-19 infection would be a serious threat to health."

Perceived benefits

1. Self-protection
- "Getting COVID-19 vaccines is a good way to protect myself from COVID-19."
2. Prevent illness in patients
- "Getting COVID-19 vaccines is a good way to prevent COVID-19 infection spread by patients"
3. Prevent illness in family or friends
- "Getting COVID-19 vaccines is a good way to protect my friends or families from infected by COVID-19."

Perceived risks and barriers

1. Acquiring the disease
- “I am afraid that COVID-19 vaccine may in fact cause me infected with COVID-19”
2. Disease from vaccination
- “I am concerned that I might have a serious side effect from a shot of COVID-19 vaccine.”
3. Vaccine unsafe
- “I am concerned that production, storage, transportation, and unprofessional injection may cause the vaccines to be unsafe for taking.”

Perceived safety and efficacy of vaccine

1. Belief that vaccine is effective
- “I am concerned that taking the vaccines might not prevent the disease?”
- “It is better to develop immunity by getting vaccine than by getting infected with COVID-19.”
2. Belief that vaccine is safe
- “I believe the COVID-19 vaccine is safe”

General attitudes and trust for vaccines

- (general attitudes) “Prevention is better than curing”
- (general attitudes) “Vaccination is effective in preventing diseases”
- (source of information) “I trust the information I receive about COVID-19 vaccines.”
- (healthcare) “I am able to openly discuss my concerns about COVID-19 vaccines shots with my doctor.”
- (healthcare) “I feel confident that the health centre or doctor's office will have the vaccine when I need to take it”
- (Politics or policies) “I trust that my government is making decision in my best interest with respect to what vaccines are provided?”
- (culture & gender) “I think COVID-19 vaccines are more important for boys/men” & “I think COVID-19 vaccines are more important for girls/women”
- (religion) “I decide to take/not to take COVID-19 vaccine because of religious or cultural reasons”
- (socio-economic) “I believe that people are risking their health or the health of the society if they do not take a COVID-19 vaccine?”
- (socio-economic) “I think it is important for everyone to get the recommended COVID-19 vaccines for themselves?”
- (geographical) “It’s important for me to spend more than one hour in travel time to get a COVID-19 vaccine”
- (industry) “I believe that vaccine producers are interested in my health.”
